# Help-seeking behavior of Jimma university students with common mental disorders: A cross-sectional study

**DOI:** 10.1371/journal.pone.0212657

**Published:** 2019-02-22

**Authors:** Yohannes Gebreegziabher, Eshetu Girma, Markos Tesfaye

**Affiliations:** 1 Department of Nursing, Debre Berhan University, Debre Berhan, Ethiopia; 2 Department of Preventive Medicine, School of Public Health, College of Health Sciences, Addis Ababa University, Addis Ababa, Ethiopia; 3 Department of Psychiatry, St. Paul’s Hospital Millennium Medical College, Addis Ababa, Ethiopia; University of Nottingham School of Medicine, UNITED KINGDOM

## Abstract

**Background:**

Globally, the mental health help-seeking behavior of university students is reported to be poor; less than one-third of university students with common mental disorders (CMDs) report having sought help from formal sources. Failure to seek treatment is associated with prolonged disability and poor mental health outcomes, including suicide. In Ethiopia, little is known about the help-seeking behavior of university students for CMDs.

**Objective:**

This study aimed to assess the prevalence and determinants of help seeking, and sources of help sought by Jimma University undergraduate students with CMDs.

**Method:**

Seven hundred and sixty students were selected to participate in this cross-sectional study using multi-stage sampling. Sources of help were identified using the Actual Help-Seeking Questionnaire. CMDs were assessed using the 10-item Kessler Psychological Distress Scale. Binary logistic regression analysis was used for both univariate and multivariable analysis.

**Results:**

Of the sampled students, 58.4% were found to have current CMDs. Of those with current CMDs, 78.4% had sought help for their problems. The majority (83.8%) of participants who sought help did so from informal sources. Compared to students who had ‘very good’ overall levels of satisfaction with life, those who had ‘good’, ‘fair’, and ‘poor or very poor’ overall level of satisfaction with life were less likely to seek help (p-value = 0.021, 0.014, and 0.011, respectively). Lastly, having no previous history of help-seeking was significantly associated with seeking help for CMDs (p-value<0.001).

**Conclusion:**

More than half of Jimma University students were found to have a high risk of CMDs and the majority of those with CMDs sought help from informal sources. Future studies are needed to explore the barriers of seeking help from formal resources, and the effects of not receiving help from formal sources for CMDs symptoms.

## Introduction

In 2015, depressive and anxiety disorders accounted for the majority of the mental disorders diagnosed worldwide [1] and often referred to as common mental disorders (CMDs). In that same year, as is reported in the reference above, an estimated 322 million people worldwide were affected by depression and 264 million people by anxiety disorders. Additionally, depression is reported to be the largest contributor to the 800,000 deaths due to suicide each year [1].

Ethiopia is one of the countries with the highest rates of depression in Africa, with a prevalence of 4.7% in the general population and more than four million Ethiopians being affected by the disorder [1]. In Africa, only Cape Verde and Lesotho reported to have higher rates of depression than Ethiopia, at 4.9 and 4.8%, respectively [1]. A systematic review of depression prevalence in Ethiopia reported a 6.8% pooled prevalence of depression from five studies which used Composite International Diagnostic Interview, and a pooled prevalence of 11.0% when three studies which used screening tools (such as Patient Health Questionnaire, and Self-reporting Questionnaire) are added in the meta-analysis [2]. Similarly, more than three million Ethiopians are estimated to be affected by anxiety disorders. Consequently, Ethiopia ranks second among African countries in terms of the proportion of total years lived with disability (YLD) attributable to depressive and anxiety disorders, at 10.1% and 3.5%, respectively [1].

University students are one of the high-risk populations for CMDs. A systematic review of depression among university students reported a prevalence rate ranging from 10% to 85%, with a weighted prevalence rate of 31% [3]. This finding was consistent with another systematic review of 24 cross-sectional studies from around the world reported a 34% pooled prevalence of depression among nursing students, with Asian nursing students reporting the highest pooled prevalence of depression at 43% [4]. Depression was found to have a prevalence of 33% in Iranian university students in a systematic review of 35 studies [5].

Students were also found to be at higher risk of pathological levels of anxiety and depressive disorders. From one study 4% of students reported having pathological levels of anxiety [6], and up to 22% of students in Sweden reported having a mental illness requiring consultation [7]. About 9% of French university students reported having major depressive disorder [8].

Certain group of students seem to be at even higher risk of CMDs like medical students. A systematic review of depression among medical students which pooled 77 studies, reporting a 28.0% prevalence of depression among medical student [9]. A more generalized systematic review including 167 cross-sectional studies and 16 longitudinal studies across 43 different countries, reported a 27.2% pooled prevalence of depression or depressive symptoms among medical students [10]. In this review, longitudinal studies showed an increase in the prevalence of depression with increases in the number of years of study. A systematic review reported a much higher prevalence of depression and anxiety among medical students in the United States (U.S.) than the age-matched controls in the general population [11]. A systematic review of studies of CMDs among medical students in English-speaking countries outside of North America reported a prevalence rate ranging from 7.7% to 65.5% for anxiety, 6.0% to 66.5% for depression, and 12.2% to 96.7% for psychological distress [12]. Similarly, a systematic review found that 11.0% of medical students in Asia reported being affected by depression [13]. Another systematic review found that Arab medical students also perceive a generally higher level of stress, depression, and anxiety [14]. However, in one comparative study with fairly representative sample, the level of anxiety in medical students are reportedly lower than that of business students [7].

When we came to Ethiopia, various studies conducted across Ethiopia reported a prevalence rate of CMDs ranging from as low as 21.6% among Adama University students and up to 63.1% among Debre Berhan University students [15–19].

If left untreated, depression can have both immediate and delayed consequences such as reduced academic performance, increased cost of treatment, and lost days at school due to disability; in extreme cases, students might terminate their education or may attempt or die of suicide [20–25]. Several studies have reported that university students have an elevated risk of dying from suicide. A systematic review of 44 studies of depressive symptoms and suicidal ideation among university students in China reported that depression increases the risk of suicide by two folds [26]. In another systematic review, about 5.8% of students reported having thoughts of hurting themselves [9]. And, a further review reported the prevalence rate of such thoughts among medical students was 11.1% [10]. Even though CMDs are a universal phenomenon associated with harmful thoughts or behaviors such as committing suicide, very few students with CMDs report seeking help.

### Help-seeking behaviour in students

Rickwood and colleagues (2005) [27] defined help-seeking as “a behavior of actively seeking help from other people” (p.4), which includes discussing one’s problem with another person to obtain support or guidance. The sources can be formal (e.g., people who have accredited professional background in the relevant field) or informal (e.g., parents and other family members).

Students’ help-seeking behavior is poor compared to the general population. A study done in the U.S. reported that only 26.9% of students with mental health conditions which require consultations, sought help from formal sources. The reported rate in the general population and aged-matched controls was 44.3% and 38.8%, respectively [28]. Various other studies support the finding that students have a low help-seeking rate from formal help sources ranging from 12.9% to 30.5% [8–10, 29–31].

A two-year cohort study among Finland high school students reported that only one-fifth of those with depression sought professional help [32]. In another follow-up study, less than half of the students reported receiving treatment for their mental health condition [33]. Additionally, students with elevated levels of depression [34], elevated levels of suicidal ideation [35], and a history of self-harm [36] are less likely to seek help than their counterparts with less serious symptoms.

The situation is more severe in Africa, as proved by a study among Nigerian students where very few students (1.5%) considered seeking help from a professional (e.g. psychiatrist or psychologist) as a recommended course of action for depression [37]. These students most commonly preferred friends and families as sources of help [37]. Despite the adverse consequences of CMDs, most students do not seek help or prefer informal sources of help than formal sources [38–41].

Factors such as fear of stigma and embarrassment, poor mental health literacy, and preference for self-reliance are the most commonly mentioned barriers to seeking help [42]. Patterns of poor help-seeking from professionals is evident even after suicidal attempt and self-harm behavior which makes the condition more complicated [43–45].

Furthermore, previous studies revealed various factors to be associated with a diminished propensity to seek help among university students including: lack of perceived need for seeking help, lack of time, lack of information about available services, low socio-economic status, male gender, preference for self-management over seeking help, and stigma [29, 46, 47].

In the context of Ethiopia, help-seeking behavior is reportedly low. One study reported that only 7% of persons with severe mental disorders living in rural communities were currently seeking help from formal sources of help (psychiatrist, psychologist, or other mental health professionals) during the study period, and just over half (56%) of people with mental health conditions had never sought help from a health facility [48]. One community-based study also reported traditional healers to be preferred over modern sources of help for mental illness [49]. Moreover, two-thirds of people with depression in another study had not sought help from any source [50]. To date, there is a lack of evidence about the help-seeking behavior of university students with CMDs in Ethiopia.

Studying the help-seeking behavior of students with CMDs is crucial for future planning to create mechanisms to help students mitigate the impact of CMDs on their lives. Thus, the aim of this study is to assess help-seeking behavior of Jimma University (JU) students showing significant symptoms of CMDs and investigate the sources of help they choose to pursue, as well as the factors associated with help-seeking from formal sources. We hypothesize that most students with CMDs will seek help from informal sources of help as opposed to formal sources.

## Methods and materials

### Study design and setting

An institution-based cross-sectional study was conducted among JU students in November 2012. JU is one of the largest public universities located in the south west of Ethiopia. During the study period, the University was made up of six main colleges namely, Engineering and Technology, Natural Science, Public Health and Medical Sciences, Agriculture and Veterinary Medicine, Business and Economics, and Social Science and Law. There were a total of 18,934 undergraduate students, of which 15,445 were male and 3,489 were female. Mental health services were available at the student clinic and JU Specialized Hospital free of charge for undergraduate students.

### Participants and sample size

The sample size for this study was calculated using a single population proportion formula at 95% confidence level, and 5% margin of error. The proportion of the condition being studied (help-seeking) was estimated as 50% as we did not find any published study on the prevalence of help-seeking for CMDs in Ethiopia. The reason that 50% was specifically chosen is to get the largest sample size, this improved the power of our study which in turn increased the quality of the study. In addition, this helped us in increasing the representativeness of the sample. The sample was drawn from the total undergraduate population of 18,934 students. Since we employed multi-stage sampling, we included a design effect of two in our calculations, to arrive at a sample size of 767, which was then further increased to 844 to account a predicted 10% non-response rate.

Multi-stage sampling was employed, which involved three stages of random selection of participants. In the first stage, groups were formed at university level using colleges as clusters. Three of the six colleges (50%) were selected randomly using the lottery method. In the second stage 50% of the departments in selected colleges were chosen again randomly using the lottery method, resulting in a total of 13 departments to be included in the study. The total sample size for the study was distributed proportionally across departments according to the number of students in each department. Within each department, the sample size was again proportionally distributed across the academic years according to class size. Finally, a systematic random sampling method was employed using students’ identification numbers (IDs) in each selected class to select study participants. The ID of the first student was selected randomly from the list of IDs from the registrar’s office, then every K^th^ individual was given the questionnaire (K was calculated by dividing the total number of students in that section by the total sample in that specific section). Class representatives from each section were contacted for ease of contacting the study participants.

### Data collection procedure

Data were collected using a structured self-administered questionnaire. The questionnaire was developed in English and translated into the Amharic language. Back translation to English was performed to ensure its consistency with the original version. The Amharic version of the questionnaire was used for the data collection.

The data collection was supervised by four trained first-year graduate students in mental health, using class representatives from the selected departments as gatekeepers. The data collection facilitators were trained for one day on how to administer the questionnaire, and how to check for completeness of the questionnaire. The questionnaire was pre-tested on 5% (n = 42) of randomly selected students, and some ambiguous words on the questionnaire were corrected before the main study. There was regular supervision of the data collection process. The data were checked for completeness and missing values every day by supervisors, and the principal investigator. Whenever missing values were found during data collection, participants were contacted again through the data collection facilitators and gatekeepers, to complete the missing data.

### Measurement of the outcome variables

#### Socio-demographic characteristics

Socio-demographic characteristics were assessed using a self-structured questionnaire, the questionnaire had seven items about sex, age, the field of study, relationship, and economic status of the students.

#### Social and clinical characteristics

Social and clinical characteristics included: the level of satisfaction with life, family history of mental illness, and social support. Level of satisfaction with life was measured using one item where participants could select a level of satisfaction on a five-point Likert scale ranging from ‘very poor’ to ‘very good’. Similarly, family history of mental illness was measured using one item with a dichotomous response (‘yes’/ ‘no’). The Three-Item Oslo Social Support Scale was used to measure social support. The scores for the scale ranged from 3 to 14. A score of 3 to 8 indicates ‘poor support’, 9 to11 indicates ‘moderate support’, and 12 to 14 indicates ‘strong support’. This Scale has been used in previous studies, there the investigators reported a good predictive validity with respect to psychological distress [51–53].

#### Mental health literacy

To measure the mental health literacy of the participants, four-items were developed by the researchers. The first item asks about having information about mental illness with a dichotomous response (‘yes’/ ‘no’). The second item investigate where they obtained information about mental illness with a list of five possible sources to tick on the one they obtained the information, students may tick in more than one sources, the third item assesses the students belief about the cause of mental illness by ticking from the four possible options, again for this item multiple response is possible, and the last item asks students awareness of the availability of mental health services within the university with a ‘yes’/ ‘no’ response.

#### Substance use

To measure substance use of the study participants, two items were developed; one item for khat (amphetamine-like substance common in Ethiopia) and the other for alcohol. The response options were structured as a five-point Likert scale ranging from ‘never’ to ‘four or more days per week’.

#### Somatic symptoms

Somatic symptoms were assessed using a three-items structured questionnaire that is adapted from the 20 items Self-Reporting Questionnaire (SRQ-20). SRQ-20 is a 20 items questionnaire, originally developed for assessment of mental distress [54]. In this section, we included three items from the SRQ-20 investigating somatic symptoms which are commonly reported by people with mental distress, namely headache, fever, and abdominal pain. Only three items were taken from the SRQ-20 for use in the present study, as we wished to include somatic symptoms that were not addressed by Kessler Psychological Distress Scale (K10), a scale we used to screen for CMDs. SRQ-20 is adapted for use in Ethiopia and validated in Amharic [55].

#### Common mental disorder (CMDs)

CMDs were assessed using the10-items Kessler Psychological Distress Scale (K10). K10 is a screening tool which investigate the respondent’s emotional state over the preceding 4 weeks. Amharic version of K10 has 10 items, each item is scored from 0 to 4 yielding a total score of 40. The K10 has already been validated for screening for CMDs in an urban Ethiopian setting. This validation study found very good psychometric properties among postnatal women in Addis Ababa with a high sensitivity (84.2%) and specificity (77.8%) at a cut-off threshold of a minimum of seven out of 40 [56]. For this study, CMDs was operationalized as scoring seven or more in the K10 scale in the four-week period prior to the study.

#### Help-seeking behaviour

Help-seeking behavior was measured using the Actual Help-seeking Questionnaire (AHSQ), which assesses recent help-seeking sources that the respondent has utilized for his/her current psychological or emotional problems in the two week period prior to the study [27]. AHSQ comprises eleven items: the first ten items list the possible sources of help, both formal and informal sources and the last item provides a choice for those who have not sought any form of help. The AHSQ score represents the number of different help sources utilized by the respondent.

Informal sources of help mentioned in the AHSQ are an intimate partner, friend, parent, other relatives/family member, minister or religious leader, traditional healer, or other help sources such as praying, reading books, and watching television. Whereas, seeking help from professional sources of help; that is, professionals who have a recognized role and appropriate training in providing help and advice, such as mental health professionals, teachers, and other health professionals are considered to be seeking help from the formal help sources [27].

Amharic adaptation of the AHSQ was not found, as a result, the AHSQ was translated into Amharic and pre-tested before the main study, although no major change was made during the pre-test. In addition, a reliability analysis of the first 10 items of the AHSQ revealed a Cronbach alpha of .60, which indicates good reliability of the Amharic version of AHSQ. For this study, mental health help-seeking is operationalized as having sought help from at least one of the help sources listed in the AHSQ, both formal and informal, to tackle their emotional problem within the two weeks preceding the study.

### Statistical analysis

Data was entered using EpiData version 3.1 software package [57] and exported to the Statistical Package for the Social Science (SPSS) version 20 [58]. The data were cleaned, and reverse coding was done for negative statements before starting the analysis. Descriptive analysis was conducted to assess inconsistencies, outliers, and missing values. The dataset was made available online through figshare.com, the Digital Object Identifier (DOI) number is 10.6084/m9.figshare.6819503.v1 [59].

The data were summarized in tables. Binary logistic regression analysis was used for both univariate and multivariable level analyses. Variables with p-values below 0.25 in the univariate analysis were considered as candidate variables for the multivariable binary logistic regression analysis. In the final model, variables with a p-value of less than 0.05 were reported to be significantly associated with seeking any form of help. The strength of association between outcome and exposure variables was described using odds ratios (OR) with 95% Confidence Intervals (CI). Crude odds ratios (COR) with 95% CI were used to describe the strength of association between outcome and exposure variable during univariate analysis. Whereas, adjusted odds ratios (AOR) with 95% CI were used to describe the strength of association between outcome and exposure variable during multivariable level analysis (adjusted for potential confounders). The study was adherent to the reporting recommendation of Strengthening the Reporting of Observational Studies in Epidemiology (STROBE) statement [60].

### Ethical statement

Jimma University Ethical Review Board approved the study (reference number RPGC/257/2012). Additional consent was sought from each department. Informed written consent was obtained from each participant. To ensure confidentiality, a code number was used instead of the participants’ names or university identification numbers, and the data were kept anonymous. Students who were found to be at risk for CMDs at screening were advised to visit either the student clinic or Jimma University specialized hospital for further assessment and treatment.

## Results

### Socio-demographic characteristics

From a total of 844 students approached to participate, 760 students completed the study, yielding a response rate of 90.1%. More than two thirds (71.2%, n = 541) of the participants, were male; the mean age of the participants was 21.16 (SD ± 1.86) years with a maximum of 30 years and a minimum of 18 years. The median monthly pocket money of the study participants was 300 Ethiopian Birr (ETB) (Table 1).

**Table 1:** Background characteristics of the study participants.

Characteristics	Responses	Frequency (n = 760)	Percentage
**Sex**	Male	541	71.2
	Female	219	28.8
**Age**	Below 20	131	17.2
	20–24	596	78.4
	25+	33	4.3
**Field of study**	Public Health and Medical Science	417	54.9
	Social Science and Law	201	26.4
	Natural Science	142	18.7
**Educational level**	1^st^ year	173	22.8
	2^nd^ year	232	30.5
	3^rd^ year	150	19.7
	4^th^ year	111	14.6
	5^th^ year and above	94	12.4
Monthly family income*	<1300 ETB	191	25.1
	1300.1–3000 ETB	274	36.1
	3000.1–4725 ETB	105	13.8
	> 4725 ETB	190	25.0
**Monthly pocket money**	<200 ETB	324	42.6
	200.1–300 ETB	186	24.5
	300.1–400 ETB	73	9.6
	>400 ETB	177	23.3
**Relationship status**	Single	618	81.3
	Other**	142	18.7

* 1 USD = 18 ETB, during the data collection period

** In relationship, married, and separated

### Social and clinical characteristics

Of the total sample, 55.7% (n = 423) reported moderate social support. More than half of the participants reported good (30.4%; n = 231) and very good (30.1%; n = 229) overall levels of satisfaction during the month of the study period. Students with poor or very poor overall levels of satisfaction accounted for 12.0% (n = 91) of the participants. Eighty-nine (11.7%) students reported having a family history of mental illness (Table 2).

**Table 2:** Social support and clinical characteristics of the study participants.

Characteristics	Response	Frequency (n = 760)	Percentage
**Social support**	Poor	193	25.4
	Moderate	423	55.7
	Strong	144	18.9
**Level of satisfaction on life**	Very good	229	30.1
	Good	231	30.4
	Fair	209	27.5
	Poor and very poor	91	12.0
**Family history of mental illness**	Yes	89	11.7
	No	671	88.3

### Mental health literacy

More than half of the study participants (52.9%; n = 402) reported that they were not aware of available mental health services at the student clinic. Three hundred and nineteen (42.0%) participants had no information at all about mental illness. Mass media (20.4%; n = 155) was the leading reported source where students obtained information about mental illness, followed by resources in the university (13.9%; n = 106). More than two third (n = 533) of the participants stated life stressors alone as a cause for mental illness, while 17.5% (n = 133) reported that mental illness may result from more than one of the stated reasons.

### Somatic symptoms, mental health condition, and substance use

One hundred forty-two (18.7%) participants reported experiencing more than one of the studied somatic symptoms (i.e., headache, back pain, or fever) within the one-month period prior to the study. However, 54.6% (n = 415) of the participants on the study reported none of the above somatic symptoms within the month prior to the study. Using a cut-off score of seven and above on the K10 scale, 58.4% (n = 444) of the study participants were at risk for CMDs during the month prior to the study. Sixty participants (7.9%) reported having thoughts of hurting themselves during the month prior to the study. Regarding substance use, 12.0% (n = 91) and 27.1% (n = 206) of the study participants used khat and alcohol more than once per month, respectively (Table 3).

**Table 3:** Somatic symptoms, mental health conditions, and substance use of the study participants.

Characteristics	Response	Frequency (n = 760)	Percentage
**Somatic symptoms**	Fever only	104	13.7
	Headache only	69	9.1
	Back pain only	30	3.9
	More than one	142	18.7
	No somatic symptoms	415	54.6
**CMDs**	Yes	444	58.4
	No	316	41.6
**Suicidal ideation**	Yes	60	7.9
	No	700	92.1
**Khat use habit**	Never	669	88.0
	Less than once a month	40	5.3
	1–3 days per month	26	3.4
	1–3 days per week	10	1.3
	4 or more per week	15	2.0
**Use of alcohol**	Never	554	72.9
	Less than once a month	133	17.5
	1–3 days per month	57	7.5
	1–3 days per week	7	0.9
	4 or more per week	9	1.2

### Pattern of help-seeking among possible CMDs cases

Among participants who are at risk for current CMDs, 78.4% (n = 348) of the students had sought some form of help for their problems, and the remaining 21.6% (n = 96) had not sought any form of help within the two-week period prior to the study. The most frequently utilized sources of help were informal help sources, which were accessed by 83.8% (n = 896) of students who had sought help. From the informal help sources, parents were consulted by 46.8% of the study participants with current CMDs, followed by an intimate partner (39.4%; n = 175), and religious leaders (36.0%; n = 160). Of those who sought help, only 16.2% (n = 173) did so from formal help sources (Table 4).

**Table 4:** Help sources accessed by students with possible current CMDs.

Help source		Frequency	Percentage	Total (n (%))
**Informal**	Parent	208	46.8	896 (83.8%)
	Intimate partner	175	39.4	
	Minister or religious leader	160	36.0	
	Friend	121	27.3	
	Other relatives/family member	121	27.3	
	Traditional healers	72	16.2	
	Other*	21	4.7	
**Formal**	Lecturers	76	17.1	173 (16.2%)
	Doctor/GP / any health care providers	66	14.9	
	Mental health professional	31	7.0	

* Praying, reading a book, watching TV, chatting with other untrained persons not specified.

One third (30.9%; n = 137) of students in the study with current CMDs reported having a history of seeking help previously, of whom 72.3% (n = 99) sought help from informal help sources. Most of these students had fewer than 11 contacts with the source they sought. The majority, 92% (n = 126), of those who had a history of previous seeking help reported that the consultations were helpful.

### Factors associated with seeking any form of help

In the multivariable level binary logistic regression analysis, it was found that the odds of seeking help was lower by 60% in those who report a good overall level of life satisfaction than those who reported a very good overall level of satisfaction (AOR = 0.4, 95% CI of 0.2–0.9, p = 0.021). Similarly, the odds of seeking help was lower by 60% in those who report a fair overall level of satisfaction in life than those who report a very good overall level of satisfaction in life (AOR = 0.4, 95% CI of 0.2–0.8, p = 0.014). Again, the odds of seeking help was lower by 70% in those who reported poor and very poor overall level of satisfaction with life, compared to those who report a very good overall level of satisfaction with life (AOR = 0.3, 95% CI of 0.1–0.8, p = 0.011). Moreover, the odds of seeking help was 6.4 times higher among those students who had no history of seeking any form of help compared to those who had sought help previously (AOR = 6.4, 95% CI of 3.0–13.5, p<0.001) (Table 5).

**Table 5:** Multivariable binary logistic regression analysis of factors associated with seeking any form of help among students with current CMDs.

Characteristics		Seek any form of help		COR (95% CI)	p-value	AOR (95% CI)	P value
		Yes (n, %)	No (n, %)				
**Sex**	Male	246 (78.8)	66 (21.2)	Ref	0.71	Ref	
	Female	102 (77.3)	30 (22.7)	0.9 (0.6,1.5)		0.9 (0.6,1.7)	0.898
**Age**	Below 20	70 (77.8)	20 (22.2)	1.0 (0.2,3.8)	0.88	0.5 (0.1,2.4)	0.425
	20–24	267 (78.5)	73 (21.5)	1.0 (0.3,3.7)		0.6 (0.2,2.5)	0.499
	25+	11 (78.6)	3 (21.4)	Ref		Ref	
**Field of study**	Natural Science	82 (86.3)	13 (13.7)	Ref	0.08	Ref	
	Public Health & Medicine	16 (76.4)	50 (23.6)	0.5 (0.3, 1.0)		0.6 (0.3, 1.2)	0.140
	Social Science & Law	104 (75.9)	33 (24.1)	0.5 (0.3, 1.0)		0.5 (02, 1.1)	0.068
**Level of satisfaction on life**	Very good	84 (90.3)	9 (9.7)	Ref	0.008*	Ref	
	Good	105 (76.1)	33 (23.9)	0.3 (0.2, 0.8)		0.4 (0.2, 0.9)	0.021*
	Fair	106 (75.7)	34 (24.3)	0.3 (0.2, 0.7)		0.4 (0.2, 0.8)	0.014*
	Poor and very poor	53 (72.6)	20 (27.4)	0.3 (0.1, 0.7)		0.3 (0.1, 0.8)	0.011*
**Belief about the cause of mental illness**	Evil (bad) spirit	24 (82.8)	5 (17.2)	Ref	0.118	Ref	
	Stress	260 (80.0)	65 (20.0)	0.8 (0.3, 2.3)		0.9 (0.3, 2.7)	0.860
	Genetic	6 (54.5)	5 (45.5)	0.3 (0.1, 1.2)		0.2 (0.0, 1.1)	0.065
	Other	11 (78.6)	3 (21.4)	0.76 (0.2, 3.8)		0.5 (0.1, 3.1)	0.485
	More than one	47 (72.3)	18 (27.7)	0.5 (0.2, 1.6)		0.5 (0.2, 1.7)	0.254
**Somatic symptoms**	Headache	37 (74.0)	13 (26.0)	0.7 (0.3, 1.5)	0.195	0.8 (0.3, 1.9)	0.605
	Back pain	15 (62.5)	9 (37.5)	0.4 (0.2, 1.1)		0.4 (0.1, 1.2)	0.113
	Fever	65 (80.2)	16 (19.8)	1.0 (0.5, 2.0)		1.0 (0.4, 2.2)	0.985
	More than one	90 (80.4)	22 (19.6)	Ref		Ref	
	No somatic symptoms	141 (79.7)	36 (20.3)	1.0 (0.5, 1.7)		0.9 (0.5, 1.8)	0.849
**Khat use habit**	Never	297 (79.0)	79 (21.0)	0.5 (0.1, 4.4)	0.232	0.4 (0.0, 3.5)	0.399
	Less than once a month	23 (79.3)	6 (20.7)	0.6 (0.1, 5.4)		0.4 (0.0, 5.0)	0.515
	1–3 days per month	14 (73.7)	5 (26.3)	0.4 (0.0, 4.1)		0.3 (0.0, 3.9)	0.380
	1–3 days per week	7 (87.5)	1 (12.5)	Ref		Ref	
	4 or more days per week	7 (58.3)	5 (41.7)	0.2 (0.0,2.2)		0.2 (0.0, 2.6)	0.218
**Previous history of help-seeking**	Yes	128 (93.4)	9 (6.6)	Ref	<0.001*	Ref	
	No	220 (71.7)	87 (28.3)	0.2 (0.1, 0.4)		6.4 (3.0,13.5)	<0.001*

* are for those variables found to be statically significant with seeking any form of help.

## Discussion

Our study found that the point prevalence of CMDs was 58.4% among JU students. This was comparable to results from previous systematic review [12]. Our finding is also comparable to previous prevalence studies conducted among Ethiopian university students [15–19]. However, our finding is much higher than the results of a systematic review which was conducted among studies of Asian students [13]. Similarly, our study is much higher than the 34.0% pooled prevalence rate of depression among students from about 24 cross-sectional studies worldwide [4]. Similar findings were also reported by other systematic reviews on which a 30.6%, 28.0%, and 27.2% weighted/pooled prevalence rate of depression among students were reported from three different systematic reviews [3, 9, 10]. Likewise, a systematic review of studies among Iranian students, reported a 33% pooled prevalence of depression, which is again lower than our study result [5].

There are various possible explanations for the higher estimated prevalence in our study, including measurement methods and timing of data collection. Firstly, this research used the K-10 scale as a screening tool for CMDs, while the other studies used a different tool. Another explanation is that since this study was conducted within a month of the students’ return from vacation for the first semester of the academic year, it is possible that students may face a number of stressors associated with moving, separating from friends and families, the admission process, changing of routine, as well as financial burden associated with fees for registration and transportation during the beginning of the semester. The presence of these added stressors at the beginning of the academic year (i.e. during our data collection period) may be a factor which explains the higher prevalence of CMDs in our study. In the future, investigators should consider data collection periods avoiding the beginning and end of the semester where students typically experience a diversity of stressors.

An important finding from this paper is that 7.9% of the participants reported having thoughts of hurting themselves during the month prior to study participation. This result is similar to that of a systematic review of 77 studies, and a study among students at Addis Ababa University, Ethiopia [9, 19]. However, it is a much higher result than found in the study done in one large public university in the U.S. [61]. This difference may be explained by a difference in the culture and socio-economic context of students of JU in Ethiopia compared to students from that large university in the U.S. For example, in Ethiopian culture, expressing one’s sadness and idea of hurting themselves is considered as a weakness, which might not be the case in the U.S. Supporting this a previous community based study in different part of Ethiopia, reported a higher degree of stigmatizing behaviour against people with mental illness [62–64]. Because of the stigma students may prefer to attempt suicide rather than explicitly look for possible solution. However, the topic of the effects of culture on suicidal behaviour might be an area for future research. Furthermore, students in Western countries may have improved access to psychologists, psychiatrists, and other mental health professionals, when compared with their Ethiopian peers.

Among the total students who are at risk for CMDs, 78.4% had sought some form of help either from formal or informal sources while the remaining 21.6% had not sought any form of help. This finding is comparable to a study conducted in the United Kingdom [65]. This is because one of the core symptoms of CMDs is hopelessness, which might further hinder the help-seeking intention of the students with CMDs. However, our finding is higher than a report from Norway [66]. The possible reason might be that we include informal sources like friends and families as a source whom students can access more easily than formal sources, but in the Norwegian study, the authors only asked the participants whether the participants ever requested help or not without elaborating the sources of help.

Another important finding is that informal sources are sought more often than the formal sources: 83.8% of all help-seeking was reported to be from informal sources of help. Conversely, only 16.2% of those who sought help visited formal help sources. In support of this, studies from Ethiopian psychiatric hospitals disclosed that majority of patients seek help from informal help sources before seeking help from modern sources and then only after a long delay (up to one year) [67–69]. Similarly, studies across different parts of the world report poor help-seeking behavior from formal help sources among students [8–10, 29–31]. However, our study reports higher help-seeking behavior rates than found in a study in Butajira, a rural district in Ethiopia [48]. This difference may be due to differences in classifications of formal help sources and differences in populations. In the definition of formal help sources, our study includes sources such as counsellors, any health professionals, and teachers, who may not have enough knowledge and practice in counselling, in addition to mental health professionals, while the Butajira study identifies mental health specialists as the only formal services. Furthermore, our sample consists of the highly educated population currently living in the city of Jimma, which may differ from the population in rural Butajira. Also, mental health services resources are easily available in Jimma compared to Butajira.

Parents, partners, religious leaders, and friends were the most frequently accessed source of help by students. Other studies have found similar results. For instance, 63.1% of participants from a United Kingdom study reported that they preferred friends or relatives as their main source of help for CMDs [65]. In addition, a study from Jimma, Ethiopia, has reported that more than half of the participants first sought help either from religious leaders or a herbalist before they visited the psychiatric hospital [67]. Again, from a study done at Amanuel Mental Hospital, Addis Ababa, 30.9% of patients first sought help from religious leaders before presenting at the hospital [68].

The finding that lower help-seeking was associated with worse overall satisfaction in life requires further investigation. It is possible that students who sought help early may have been recovering from their CMDs symptoms. However, causal associations cannot be drawn from this cross-sectional data. The other variable found to have an association with seeking help was a history of seeking help. According to this study, the odds of seeking help were six times higher in those students who had no previous history of help-seeking. It is possible that those who had previously sought help for similar conditions were not satisfied with the care they received, thus were less likely to seek help again. This finding warrants further exploration so that a culturally appropriate and acceptable mental health service strategy can be developed.

This study is the first study in Ethiopia regarding help-seeking behavior for CMDs in university students. Previously there have been three studies on pathways to care for mental illness in Ethiopia, which reported that most patients with a mental health condition sought help from traditional treatment places before eventually visiting the mental health hospitals, after a long delay [67–69]. There are additional studies which investigated help seeking, though those studies suffered from selection bias as they only recruited patients who presented to the hospital for treatment. Persons with CMDs were underrepresented in those samples. In addition, none of those studies explored the help-seeking behavior of students with probable CMDs. This study assessed the prevalence of CMDs and both formal and informal sources of help that the students sought during two weeks prior to the survey.

Despite those strengths, this study has several limitations. First, information about help-seeking and CMDs status was based on self-reported data, which is subject to several biases, primarily recall bias and social desirability bias (pressure to give a socially desirable response). Thus, we had no opportunity to confirm students’ responses with other informants living with them. Second, the cross-sectional design does not allow attribution of causality. Third, the study was conducted at the beginning of the semester when high levels of stress among students may be expected, which may affect the findings of this study. For future studies, it may be preferable to conduct similar studies at different points of the academic year. Fourth, even though we have used the K10 scale, which was validated for the use in Ethiopian settings, the instrument was only validated to screen for CMDs among clients in the antenatal clinics in Ethiopia. However, the sensitivity and specify from the original validation study suggest that the tool is effective in measuring the prevalence of probable CMDs. The internal consistency of the measure in our sample was high as measured by a Cronbach’s alpha (0.89). Fifth, the concept of help-seeking is broad and this study explores only certain aspects of help seeking. For example, barriers and facilitators of seeking help are not addressed. Finally, since all the participants were students at Jimma University, our findings may not be generalizable to other communities in the country, particularly those outside the university settings.

## Conclusions

The prevalence of CMDs among Jimma University students was estimated to be 58.4%. Among those at risk for CMDs, 78.4% had sought some form of help either from formal or informal sources; the remaining 21.6% had not sought any form of help. Only 16.2% of all those seek help sought help from formal help sources, while the majority (83.8%) participants who sought help, did so through informal sources. Parents, partners, religious leaders, and friends were the most frequently visited sources of help by the study participants.

The findings of this study highlight that additional interventions are needed to improve the help-seeking behaviors of students with CMDs and direct them towards formal help sources. Future studies are needed to explore the barriers to accessing formal mental health services experienced by university students. In addition, longitudinal studies focusing on the impact of not seeking help might be a promising area for future researchers.
